# Enhanced thermomechanical properties of epoxy-multiwalled CNT nano-composites†

**DOI:** 10.1039/d4ra06831a

**Published:** 2024-11-05

**Authors:** Abdullah Alhendal

**Affiliations:** a Department of Chemistry, Kuwait University P. O. Box 5969 Safat 13060 Kuwait Abdullah.alhendal@ku.edu.kw

## Abstract

Viscoelastic properties of thermo-set composites using an epoxy matrix reinforced with pristine CNT and silane-modified MWCNT at different concentrations (0%, 1%, 2% and 4%) were studied to observe the enhanced thermal and mechanical properties supplemented by the increased interfacial interaction due to CNT modification. The composite with pristine CNT was labeled as EPB-CNT, whereas that with silane-modified carbon nanotubes (CNTs) was referred to as ECB-CNT. The silanes used were glycidyloxypropyltrimethoxysilane (GPTS) and 3-aminopropyltriethoxysilane (APTES). Diglycidyl ether of bisphenol-A (DGEBA) was completely cured by Jeffamine D-400 to prepare EJ-0. The amine groups of the 3-aminopropyltriethoxysilane (APTS) partially cured the diglycidyl ether of bisphenol-A (DGEBA) in EAJ-0 by a sequential polymerization process, while the methoxy groups subsequently produced a silica network through the sol–gel method. Subsequently, Jeffamine D-400 was used as a curing agent at elevated temperatures for cross-linking and complete curing. EJ-0 and EAJ-0 were considered as neat films of EPB-CNT and ECB-CNT composites, respectively. Tensile and storage modulus tests, thermal property analysis using TGA, and microstructure characterization using field emission scanning electron microscopy (FESEM), atomic force microscopy (AFM), and TEM were all part of the study. Comparing composites with varying percentages and with neat films, the chemically bonded epoxy-silanized MWCNTs (ECB-CNTs) showed improved performance. ECB-CNT 4% had the highest tensile and storage modulus as well as improved thermal stability. Improved filler material distribution and fewer voids were found through microstructure analysis, strengthening the link between the reinforcement and matrix. The results underscore the potential applications of the CNT-enhanced nanocomposites in the engineering fields of automotive, aerospace, radar-absorbing materials and others. This marks a significant development in the field of composite technology to produce durable and effective materials.

## Abstract 2

(A) Preparation of (GPTS-APTS) modified MWCNT solution/GA-CNT, (B) preparation of sol–gel active and partially cured polymer matrix and (C) preparation of ECB-CNT composite.

## Graphical abstract

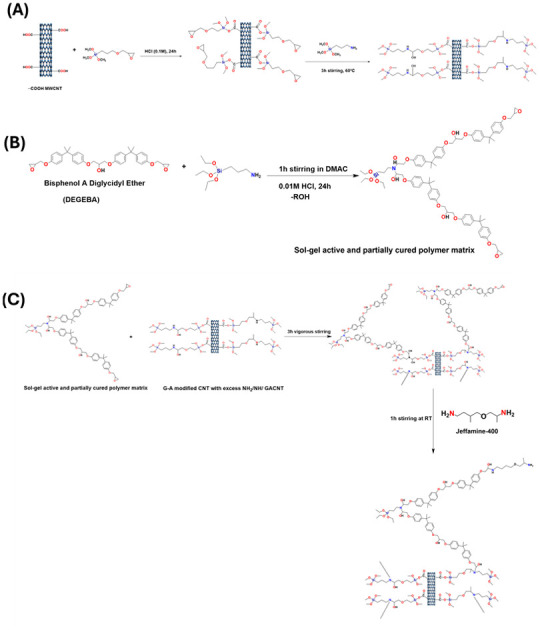

## 1. Introduction

Functionalized multiwalled carbon nanotubes (MWCNT) are used as reinforcement in polymer matrices due to their distinctive mechanical and thermal properties, achieved through structural modification.^1–4^ The properties of these composites depend not only on these structural changes but also on various factors, such as the aspect ratio of the tubes used, homogeneity in its dispersion without aggregation, and interaction of carbon nanotubes (CNTs) with the polymer matrix. Both aggregated CNTs and broken tubes in the polymer matrix will reduce the thermal and mechanical stability. However, the nanoscale dispersion of fillers without tube breakage creates a large interfacial interaction between the fillers and the polymer matrix chains, which will improve the mechanical properties of the composites.^3^ The dispersion of fillers in linear polymer chain matrices may not be significantly affected by the system viscosity. However, in branched polymer chains, such as in thermo-set polymers, where both organic and inorganic networks form concurrently, the viscosity of the system can greatly affect the filler distribution and can hinder the development of one phase in the presence of the other.^5,6^ The use of a CNT filler in polymer composite preparation can offer exciting opportunities in various fields of science and technology.^7^ Owing to the significant tendency of the inter-tube van der Waals interactions toward agglomeration, a uniform dispersion of CNTs in the matrix is crucial. Thus, this calls for their stabilized de-aggregation inside the matrix.^8^ Amit Kumar *et al.* conducted a thorough observation on CNT dispersion in the epoxy matrix, and proposed that the selection of the hardener also will affect the CNT dispersion *via* properties like temperature and viscosity.^9^ Ultrasonication, calendering, and ball milling are useful at low concentration of CNTs. However, at high concentrations, the increase in the resin viscosity can make their dispersion difficult. Surface treatments of CNTs by physical and chemical methods can also be used to enhance the interfacial interaction with the matrix,^10^ which will change the nature of the resulting interphase and thus potentially improve the physical, thermal, mechanical, and electrical properties of the composites.

Silanization,^2,11,12^ amino-functionalization^13,14^ and acid-functionalization^12,15^ reactions are the most suitable ways to improve the CNT compatibility and the dispersion within the polymeric matrix. The surface chemical modification of CNTs is generally made through preventive oxidation processes, which induce the formation of acidic and hydroxyl groups on the surface, binding several organic moieties on the surface.^16^ It creates several defects on the sidewalls and on the end tips, and leads to the consequent breaking of the CNTs. This results in the reduction of the aspect ratio, which is normally inimical for the mechanical properties of nanocomposites.^13,17^ However, silanization of CNTs has been shown to be a fruitful approach for decreasing the reduction of the CNT aspect ratio resulting from chemical treatments. Organosilanes have two reactive end groups: one hydrolysable group (Si-OR) that can condense with hydroxyl functionalities present on the MWCNT surface, and the other functional group which can react with the polymer matrix material or another suitable silane material. Ma PC *et al.*^18^ and Velasco-Santos *et al.*^11^ have studied the silanization of MWCNTs by using 3-glycidoxypropyltrimethoxysylane and organosilanes. Kathi *et al.*^19^ and Lee *et al.*^20^ showed that the MWCNTs functionalized with 3-aminopropyltriethoxysilane enhanced the thermal and mechanical performances of epoxy-based composites. Danny *et al.*^21^ observed the effect of the silane structure on the properties of the silanised MWCNT-epoxy composites, and Jean Carlos *et al.*^22^ investigated the effect of the MWCNT/GLYMO ratio in the preparation of dispersible reinforcements for epoxy matrices. Liu *et al.*^12^ conducted a study to understand the structural effect of hyperbranched siloxanes on the thermal stability of polyurethane composites.

Due to the 3D crosslinking network formed by the hardener, epoxy resins are counted as a highly reactive polymer that can be stiffened by nanoparticles. They are superior in adhesion, corrosion/heat resistance and mechanical properties and are widely used as coatings, adhesives, varnishes, encapsulations, mouldings, *etc.*, in automotive, aerospace and civil construction industries. Attempts have also been made to improve the mechanical and thermal properties of such polymeric systems by the addition of inorganic networks. Solvents like acetone and THF have been previously used To overcome the high viscosity problem. However, as the cross-linking and the condensation of the silica network in the sol–gel process take place at a higher temperature, the low-boiling solvents were not effective in controlling the morphology. Arash *et al.*^23^ have studied the viscoelastic properties of multi-walled carbon nanotube/epoxy composites using two different curing cycles. Two different curing cycles have been studied, 4 h at 80 °C, and the other curing cycle involved 24 h at room temperature and post-curing 4 h 80 °C. It is reported that the effect of nanotubes is more prominent for a resin that was cured at a lower time. Generally, epoxy resins are completely inert once they are fully polymerized, and thus environmentally safe. Epoxy resin is generally non-toxic and poses minimal threats if accidently ingested, touched, or inhaled. The volatile emissions from our epoxies are much lower than that of conventional vinyl esters and polyesters used in the composites industry, and so they are essentially safe materials when using in a cured state.^24^

In the present work, we have used dimethylacetamide (DMAC) as a solvent. DMAC evaporates steadily, and enhances the crosslinking and condensation of inorganic–organic phases. The sol–gel technology was employed to prepare the ECB-CNT composite material. Afterwards, Fourier-transform infrared spectroscopy (FTIR) and X-ray photoelectron spectroscopy (XPS) were employed to identify the structure of the hybrid material. An ultrathin film was prepared separately for FTIR. Due to the slight increment in the thickness of the hybrid film, XPS was also used. The thermal stability and mechanical strength of the developed hybrid materials were examined using thermogravimetric analysis (TGA) and the dynamic mechanical and thermal analysis (DMTA). Various heating protocols were tested (*e.g.*, heating at 100 °C for 4 hours; heating at 80 °C for 6 h, and then 100 °C for 3 h; heating at 80 °C for 18 h, and then heating at 120 °C for 2 h; heating at 100 °C for 3 h, and then at 120 °C for 3 h) to complete the sol–gel process and the reaction between the amine groups from Jeffamine-D-400 and glycidyl groups on the DGEBA. FTIR analysis was used to discover the best curing protocol. The heating for 18 h at 80 °C and then at 120 °C for 2 h was found to give the best results. Earlier heating at higher temperature was responsible for the evaporation the monomers and led to incomplete curing, whereas the heating at 120 °C for a longer time also caused the degradation of epoxies as the films turned yellow in colour.

## 2. Experimental

### 2.1 Materials and instrumentation

Diglycidyl ether of bisphenol-A with a molar mass of 340.41 g mol^−1^ (DEGEBA, equivalent weight per functional group: 170.21) was obtained from Sigma Aldrich, USA. Jeffamine-D-400 (molar mass: 430 g mol^−1^) was obtained from Huntsman. Aminopropyltriethoxy silane (APTS, 98%, molar mass: 221.37 g mol^−1^), (glycidyloxypropyl) trimethoxysilane (GPTS, 98%, molar mass: 236.34 g mol^−1^), acid-functionalised MWCNT (–COOH MWCNT, ID: 2–5 nm, OD: 7 nm and length 10–20 nm, US Research Nanomaterials) and *N*,*N*-dimethylacetamide (DMAC) (≥99.5%) were obtained from Sigma Aldrich. All the chemicals were used as received.

The pure epoxy and composite films with various CNT loadings were characterized using different techniques as described earlier. FTIR absorption spectra for the neat epoxy and epoxy-silica hybrid films in the range of 400–4000 cm^−1^ were taken by the JASCO FTIR spectrometer to determine the optimum curing parameters of the epoxy resin, CNT bonding, and the nature of the silica produced. Thin films were prepared and cured under the same procedure as given above for this analysis. XPS was used to determine the surface elemental composition of C, O, Si and N to identify the functional groups present. The XPS spectra were recorded with a Thermo ESCALAB 250 Xi using a monochromatic radiation source (1486.6 eV) with a spot size of 850 micrometers. Spectra acquisition and processing was carried out using the software Thermo Advantage Version-4.87. The base pressure in the XPS analysis chamber was kept in the range of 10^−9^–10^−10^ torr. The analyser was operated with a pass energy of 20 eV, and a dwell time of 50 min with a step size of 0.1 eV. The morphology and the dispersion of the MWCNTs and silica were investigated using a scanning electron microscope (SEM) that was conducted in high-resolution mode using a JEOL-JSM-7001F instrument operated at 25.0 kV. The surface morphology was examined by atomic force microscopy (AFM). Topographic AFM images of the samples were obtained by a Nano-scope IV Scanning Probe Microscope using contact AFM in air in the constant force mode (1 ± 2 nN). Images were captured with the tapping mode “RTESP” tips. The cantilever is a gold-coated silicon nitride one with a silicon tip of 20 nm radius. An area of 5 μm was scanned. The background was subtracted from the images using nano scope software. Dynamic mechanical and thermal analysis was carried out using DMA Q-800 (TA, USA). The measurements of the storage modulus and glass transition temperature (*T*_g_) were made under tension mode in the temperature range from ambient to 140 °C at a heating rate of 2 °C min^−1^ using a frequency of 1 Hz under an inert atmosphere. Thermogravimetric analysis was performed on epoxy-CNT hybrid systems by measuring the change in the weight of the samples with increasing temperature using the Shimadzu TGA-50 analyzer. Approximately 10 mg of the sample was heated from ambient temperature to 800 °C at a heating rate of 10 °C min^−1^ in static air. The temperature for the maximum decomposition was measured from the first differential of the TG curves. Tensile testing was performed at room temperature on the Shimdzu Autograph TRAPEZIUM X with a strain rate of 5 mm min^−1^, according to ASTM D 882 (standards).

### 2.2 Synthesis of the epoxy neat film cured by Jeffamine-D-400 (EJ-0)

Equivalent amounts of DEGEBA and Jeffamine-D-400 were mixed in DMAC in a 100 mL bottle and stirred for 1 h. The resulting mixture was subsequently poured into Teflon Petri dishes, and left to cure for 18 hours at 80 °C, followed by 1 hour at 120 °C without vacuum and 1 h at 120 °C under vacuum.

### 2.3. Synthesis of the EPB-CNT composites

DEGEBA in DMAC was added to calculated the amounts of pristine MWCNT solution, and stirred vigorously for 4 hours at 60 °C. The temperature was then lowered to 25 °C, and an equivalent amount of Jeffamine-D-400 was added. The mixture was then agitated for an additional hour, adhering to the same casting and curing conditions as the neat film EA-0 of the EPB-CNT system. Table 1 summarises the preparation of the EPB-CNT composites for different CNT wt%.

**Table 1:** Composition of EPB-CNT composites and variation of the glass transition temperature (*T*_g_)

Sample name	DEGEBA (wt in g)	DMAC (mL)	MWCNT-P (wt in g)	Jeffamine-D-400 (wt in g)	*T*_g_ (°C)
EJ-0	1.8387	3	NIL	1.1613	58.74
EPB-CNT-1%	1.8203	3	1.83	1.1496	59.07
EPB-CNT-2%	1.8019	3	3.66	1138	61.73
EPB-CNT-4%	1.7651	3	7.32	1.1148	63.06

### 2.4 Preparation of the epoxy film cured by Jeffamine-D-400 and APTS (EAJ-0)

In the synthesis, DEGEBA and calculated amounts of APTS for 50% NH_2_ were dissolved in the solvent DMAC and stirred vigorously for 30 minutes at 60 °C. APTS and water were mixed in a 1 : 2.25 molar ratio. 0.05 M HCl was added, and the mixture was stirred for another 4 h at 60 °C to complete the sol–gel process. The temperature was reduced to 25 °C, and then a calculated amount of Jeffamine-D-400 was added to the reaction mixture before stirring again at 25 °C for 1 hour. Finally, the mixtures were poured into Teflon Petri dishes, and cured under the same conditions as the EJ-0 film.

### 2.5 Preparation of ECB-CNT composites

**Fig. 1:**
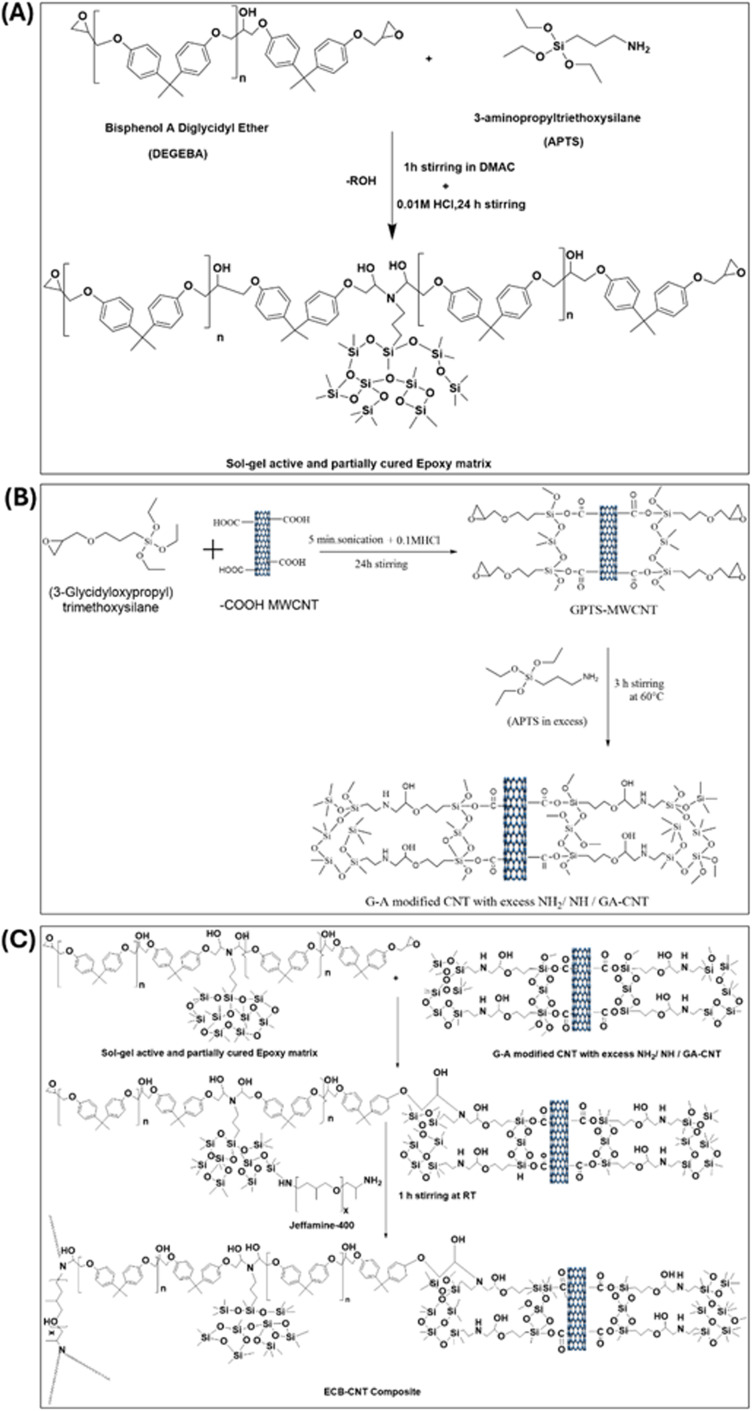
(A) Preparation of (GPTS-APTS)-modified MWCNT solution/GA-CNT. (B) Preparation of the sol–gel active and partially cured polymer matrix, and (C) preparation of the ECB-CNT composite.

Fig. 1 provides the stepwise synthetic routes for the preparation of the chemically bonded, epoxy-modified MWCNT composite system. The preparation is also summarized in Tables 1–3.

**Table 2:** Composition of the GA-CNT solution

MWCNT (wt in g)	DMAC (mL)	GPTS (wt in g)	0.01 M HCl (wt in g)	APTS (wt in g)
			APTS : H_2_O – (1 : 2.25)	
0.3	18	0.6122	0.1028	0.5734

**Table 3:** Composition of ECB-CNT composites and variation of the glass transition temperature (*T*_g_)

Sample name	DGBA (wt in g)	Proportions of curing agents (wt in g)		GA-CNT (wt in g)	DMAC (mL)	*T*_g_ (°C)
		Jeffamine-D-400	APTS			
EA-0	1.8387	0.5807	0.6097	NIL	3	74.03
ECB-CNT-1%	1.7389	0.5114	0.5368	1.9473	3	76.86
ECB-CNT-2%	1.6392	0.4829	0.4163	3.8946	3	78.86
ECB-CNT-4%	1.4397	0.4234	0.3536	7.7892	3	78.68

#### 2.5.1 Preparation of the (GPTS-APTS)-modified MWCNT solution/GA-CNT

0.3 g of –COOH MWCNT was dissolved in 18 g of DMAc and subjected to sonication for 3 hours, followed by stirring for 24 hours. 0.6 g GPTS was added to this CNT solution, which was then sonicated for 5 minutes. Next, a calculated amount of 0.01 M HCl was added and stirred for 24 h. Subsequently, APTS was added to cure the epoxide terminal groups of GPTS. APTS was used in excess to ensure that the modified CNT retained unreacted amine groups, which could then react with DEGEBA to form a bond between the polymer and the modified CNT.

#### 2.5.2 Preparation of the sol–gel active and partially cured polymer matrix

To prepare the sol–gel active polymer matrix, DEGEBA and calculated amounts of APTS for different –NH_2_% were dissolved in the solvent DMAC and stirred vigorously for 1 hour at 60 °C in 100 mL bottles. To complete the sol–gel process, 0.05 M HCl was added in a 1 : 1.25 molar ratio relative to APTS, and the mixture was stirred for 3–4 hours at 60 °C. This process utilizes the amine end groups of APTS to cure approximately 50% of the epoxide groups of DEGEBA.

#### 2.5.3 Preparation of the ECB-CNT composite

To prepare the composite films, calculated amounts of the GA-CNT solution were added to the sol–gel active polymer matrix and stirred for another 2 to 3 hours at 60 °C. The temperature was then reduced to 25 °C and the required amount of Jeffamine-D-400 was added to each reaction mixture, followed by stirring again at 25 °C for 1 hour. Finally, the mixtures were poured into Teflon Petri dishes and cured under the same conditions.

## 3. Results and discussion

### 3.1 Fourier transform infrared spectroscopy (FT-IR)

**Fig. 2:**
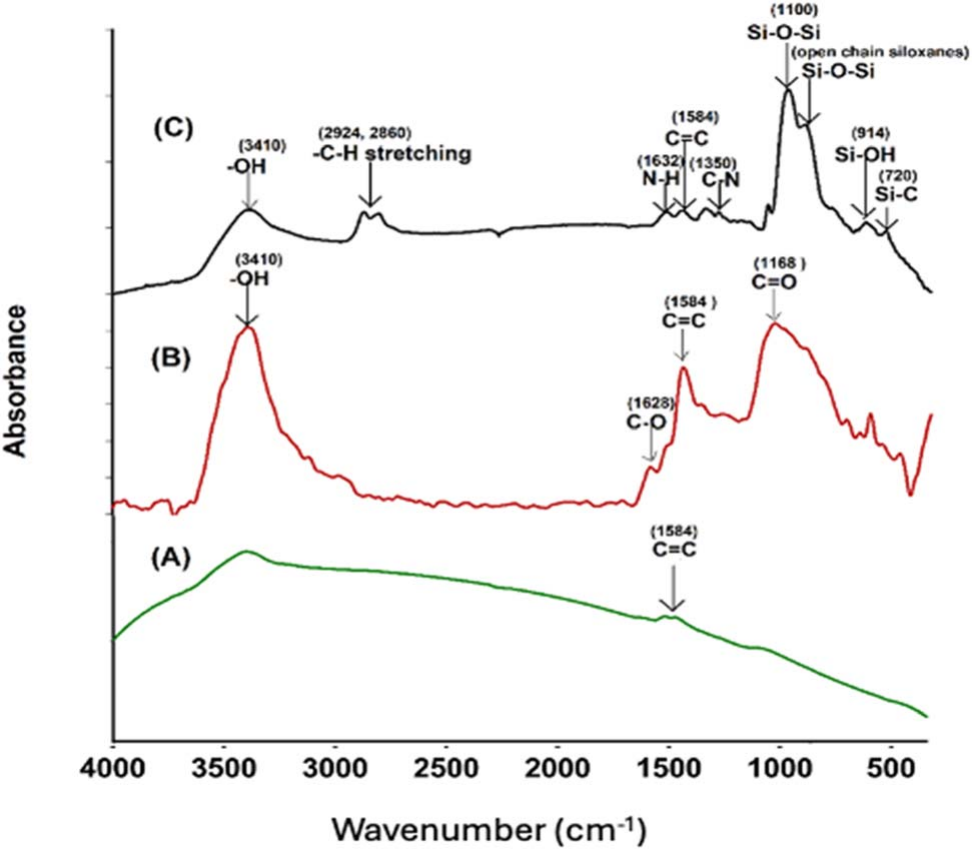
FTIR spectra of (A) MWCNT, (B) –COOH CNT, and (C) GA-CNT.

Successful surface modification of the –COOH MWCNT with GPTS and APTS was evidenced by FT-IR spectra. FT-IR analysis of the pristine MWCNT, –COOH CNT, and GA-CNT was performed to verify possible structural differences in these samples, and the spectra are shown in Fig. 2. All the intensities of the peaks corresponding to the oxygen-containing acid functional group were observed to increase, as compared to the intensities of the peaks of pristine MWCNT. In the FT-IR spectrum of the modified CNT, silica peaks were clearly visible at 1100 cm^−1^.^25^ Successful surface modification of acid-functionalized CNT by the silanes was confirmed by FT-IR spectra.^26^ The peaks due to the presence of carboxylic groups disappeared after silanization by GPTS, indicating the reaction of the carboxylic groups with the alkoxy group of GPTS. The peak at 3410 cm^−1^ in the GANT became broader and weaker, the C=O peak at 1380 cm^−1^ disappeared, and two small bands due to the C–H stretching of the methylene groups formed at 2860 cm^−1^ and at 2924 cm^−1^.^18^ The epoxide group from GPTS then reacted with amine from APTS. The peaks at 1350 cm^−1^ and 1632 cm^−1^ can be attributed to the C–N stretching and N–H bending of the amine group of the silane, respectively.^12^

**Fig. 3:**
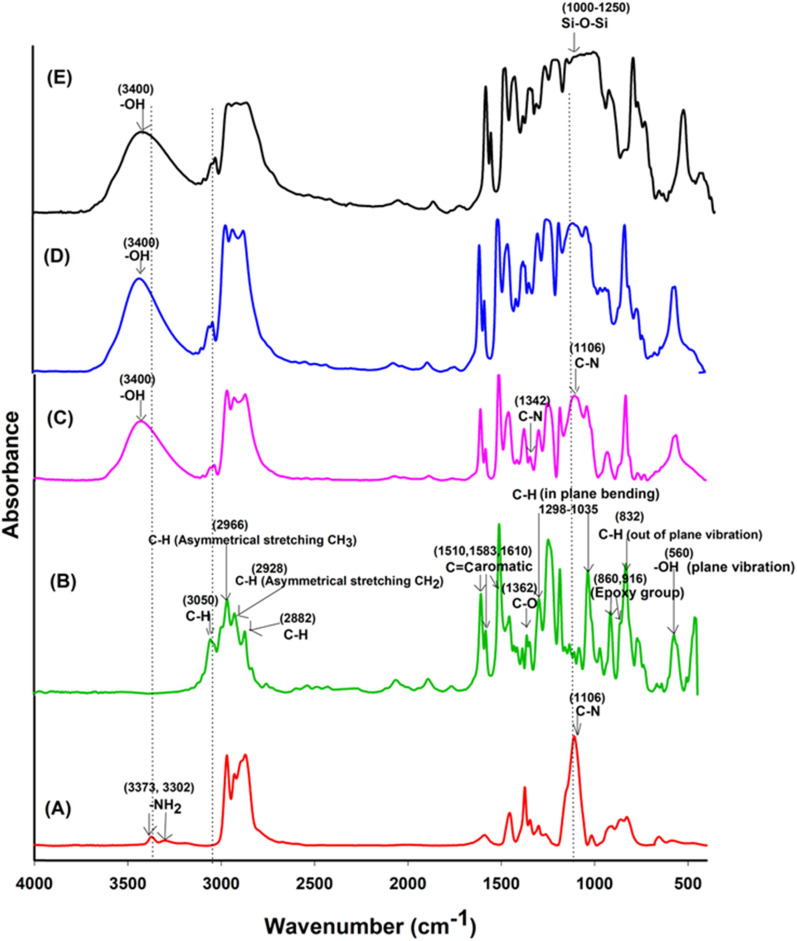
FTIR spectra of (A) Jeffamine-D-400, (B) uncured DEGEBA, (C), EA-0, (D) EAJ-0 and (E) ECB-CNT-4% Composites.

In Fig. 3, a comparison of the FTIR spectra for uncured Jeffamine-D-400 (A), uncured DGEBA (B), EJ-0 (C), EAJ-0 (D), and ECB-CNT-4% (E) is shown. Spectra (B) and (C) in Fig. 3 depict the FTIR of the uncured and cured DEGEBA, respectively. The curing of epoxy resins results in the formation of the OH group (stretching) due to the epoxy ring opening, which appears as a broad peak in the range of 3200–3600 cm^−1^ in cured DEGEBA and in the hybrid films. The curing of epoxies with diamine is a two-step process, which resulted in the formation of a tertiary amine peak at 1106 cm^−1^, suggesting the cross-linking reaction of the amino groups with epoxide. The disappearance of epoxy groups, as indicated by the oxirane ring peaks at 915 cm^−1^, 860 cm^−1^ (asymmetrical stretching), and 3050 cm^−1^ (C–H symmetric stretch in the epoxide) from the uncured DEGEBA spectra, is attributed to the ring-opening reaction.^27^ A small peak at 1342 cm^−1^ reveals the formation of the tertiary amine symmetric stretching in the cured DEGEBA, which again confirms the successful curing. The disappearance of the aliphatic primary amine groups at 3373 cm^−1^ and 3302 cm^−1^, along with the formation of –OH groups in the cured films and the presence of a tertiary amine peak at 1106 cm^−1^, reveals the cross-linking reaction of the amino groups with epoxide. In Fig. 3(D), APTS was used for the partial curing of DGEBA. Hence, the silica network developed by sol–gel reaction also is present at 1106 cm^−1^.^27^ In the cured DEGEBAs and in all hybrid films, the absorption peak around 3036 cm^−1^ is due to CH=CH. The peaks at 2976 cm^−1^, 2928 cm^−1^ and 2872 cm^−1^ are due to the asymmetric and symmetric stretching modes of the CH_2_ groups, and are from the epoxy matrix. The absorption peak for Si–O–Si asymmetric stretching appears in the range of 1000–1250 cm^−1^, but as a flat and broad peak in the spectrum of the ECB-CNT-4% composite. Table S1† gives the FT-IR frequencies of the DEGEBA *vs.* the cured DEGEBA.

### 3.2 X-ray photoelectron spectroscopy (XPS)

**Fig. 4:**
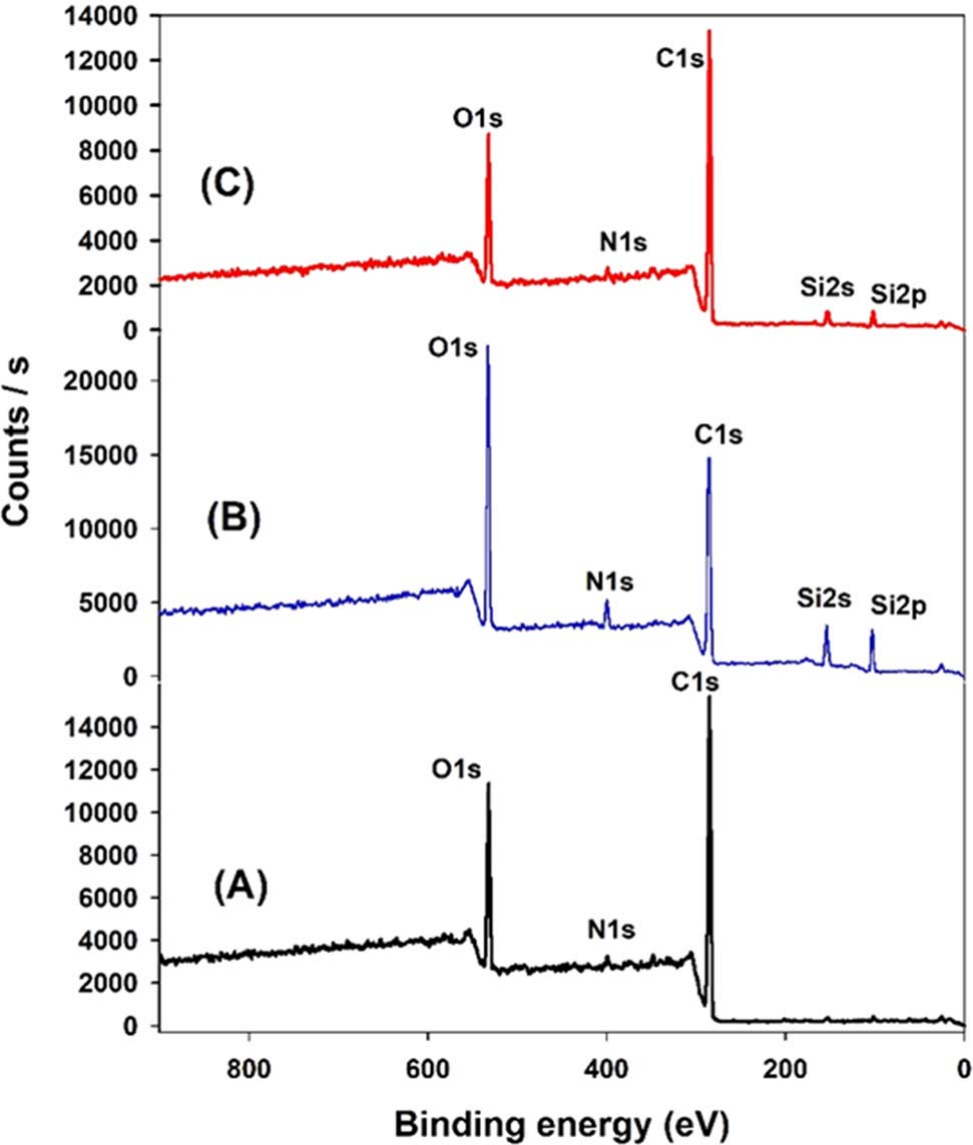
XPS survey of (A) EA-0, (B) GA-NT, and (C) ECB-CNT-4% composites.

**Fig. 5:**
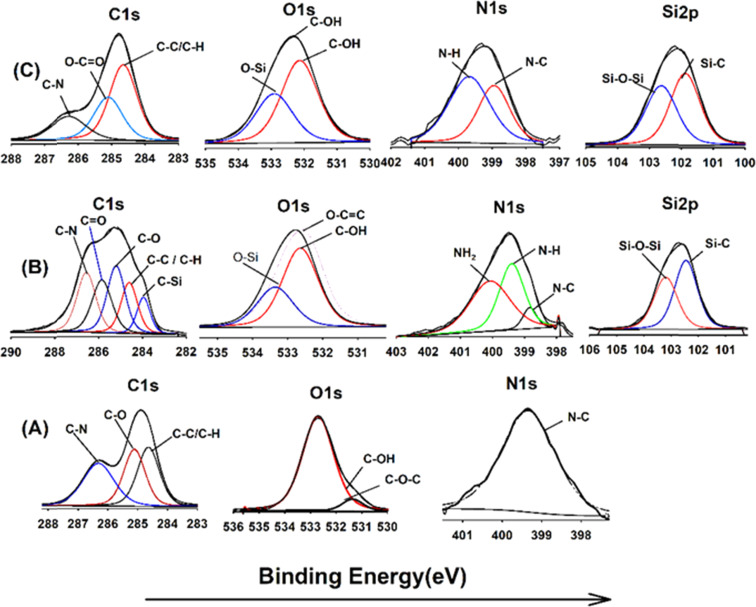
XPS spectra of (A) EA-0, (B) GA-CNT and (C) ECB-CNT-4% composites.

XPS analysis was employed to elucidate the surface composition of EA-0, GA-CNT, and ECB-CNT-4% composites, providing insights into the interactions between MWCNTs and silane coupling agents, as well as the bonding of GA-CNT within the epoxy polymer matrix. Fig. 4 and 5 show the XPS survey and deconvoluted peaks of EA-0, GA-CNT and ECB-CNT, respectively, to describe the chemical bond formation in the ECB-CNT hybrid film. In EA-0, no silica peaks were detected as expected. However, in GA-CNT and ECB-CNT-4%, Si–C and Si–O–Si peaks are observed due to the presence of silanes.^28^ The peak at 531.36 eV corresponds to C–O–C ring carbon bonding, which is present in a small amount in EA-0.^29^ In all composites, the C1 peak located at 284.6 eV corresponds to the C–C/C–H group, while another peak at 285.3 represents the C–O group. The C–N peaks in EA-0 and ECB-CNT-4% were observed at 286.3 eV, whereas, it is found at 286.5 eV in GA-CNT. The atomic% of the C–C peak in ECB-CNT-4% is found to be increased considerably from EA-0 due to the incorporation of modified CNT. The deconvolution of the O 1s spectrum of ECB-CNT-4% gives two peaks at 532.14 eV and at 532.92 eV, which are attributed to C–OH and C–Si, respectively.^30^ The C–Si peak was also present in GA-CNT, providing strong evidence of successful silanization.

The deconvoluted N 1s peak of the XPS spectra of EA-0 gives a solitary peak at 399.3 eV, which can be ascribed to the *tert*-amine (N–C) formed by the curing of DEGEBA. It also substantiates the FT-IR results given in Fig. 3, which clearly points out the disappearance of the primary amine and the formation of a *tert*-amine in the cured films. The primary amine peak (NH_2_) seen in GA-CNT, due to excess APTS, disappeared in ECB-CNT-4% because of the effective incorporation and cross-linking of GA-CNT in the epoxy matrix. Consequently, only the N–H peak is observed in the ECB-CNT hybrid, which is slightly shifted to a lower binding energy value corresponding to the N–H peak.^31^

### 3.3 Atomic force microscopy analysis (AFM)

**Fig. 6:**
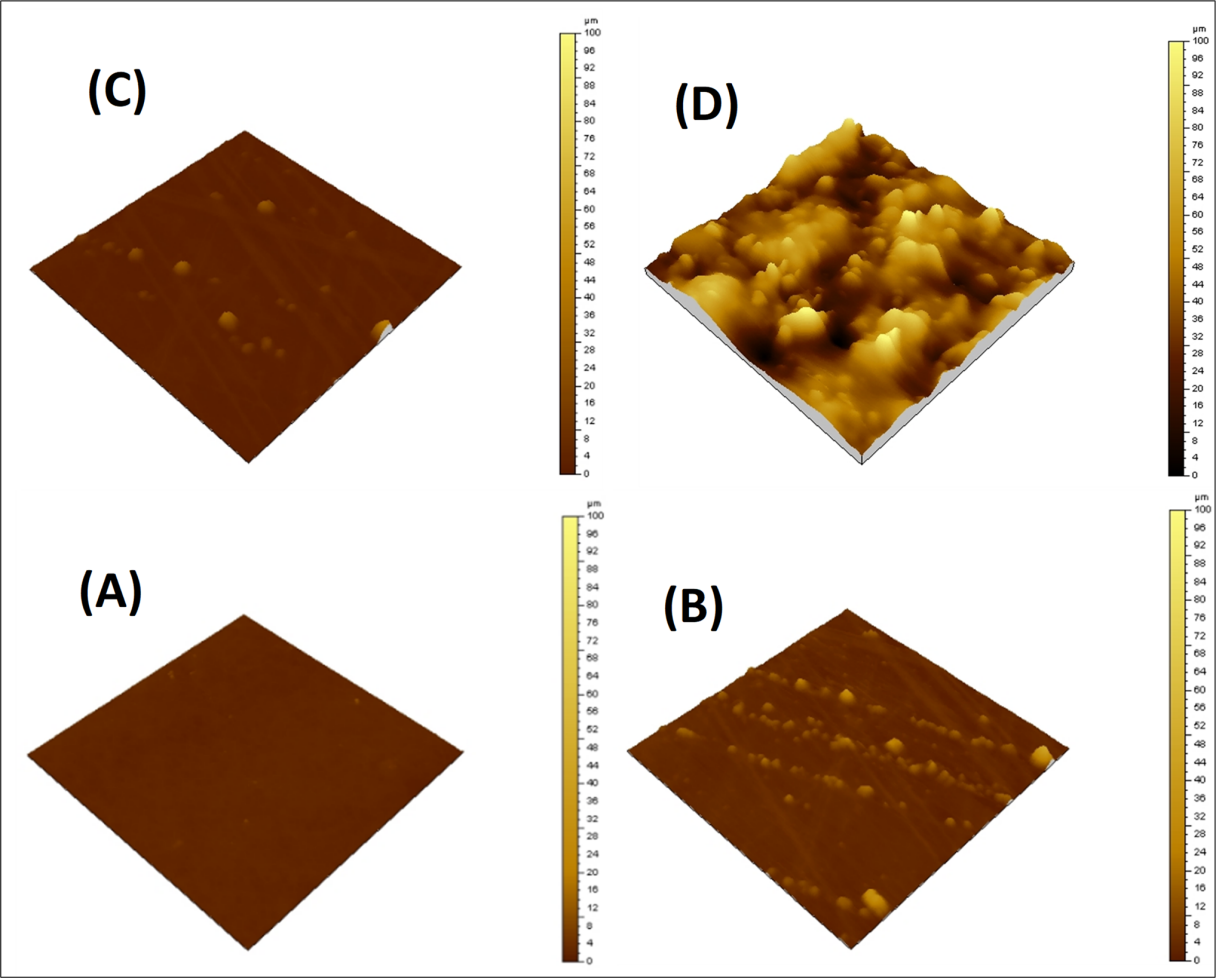
AFM images of the cured epoxy films with different CNT loadings; (A) EA-0, (B) EPBCNT-4%, (C) EAJ-0 and (D) ECB-CNT-4% composites.

Surface morphology of the neat matrix and its composites with the siloxane-modified CNT network was evaluated by AFM. A flat and smooth surface was observed for EJ-0, with no visible defects. The AFM image shows a little more surface roughness in EAJ-0 due to the presence of silica derived from APTS. In ECB-CNT-4%, the film surface roughness is increased due to GA-CNT incorporation and can be seen by the AFM images from Fig. 6(D). The lighter regions represent the inorganic phase, and the phase contrast reveals localized areas of soft and hard materials on the surface. The AFM image of the EJ-0 film is defect-free, whereas the DEGEBA cured by the partial replacement of Jeffamine-D-400 by APTS (EAJ-0) shows a slightly rough surface due to the silica network structure. The surface roughness increased significantly in the ECB-CNT-4% hybrid due to the inclusion of modified CNTs, which contain silica from both APTS and GPTS, along with interdiffused polymer and MWCNT. This increased the compatibility between the epoxy matrix and GA-CNT. As shown in the AFM image in Fig. 6, the silica network agglomerates into discrete particles, and chemical bonding occurs between the organic part of the silane and the epoxy network. This leads to the development of organic–inorganic networks in ECB-CNT-4%. The APTS cured the epoxide ring from both GPTS and that from DEGEBA, and developed the silica network. Thus, the (GPTS-APTS)-functionalized MWCNT formed a hyperbranched structure, causing increased interfacial adhesion and a well-defined hybrid morphology. This facilitates the stress transfer from the matrix to reinforcement under stress, and thus improves the mechanical strength.^32^

### 3.4 Scanning electron microscopy (SEM)

**Fig. 7:**
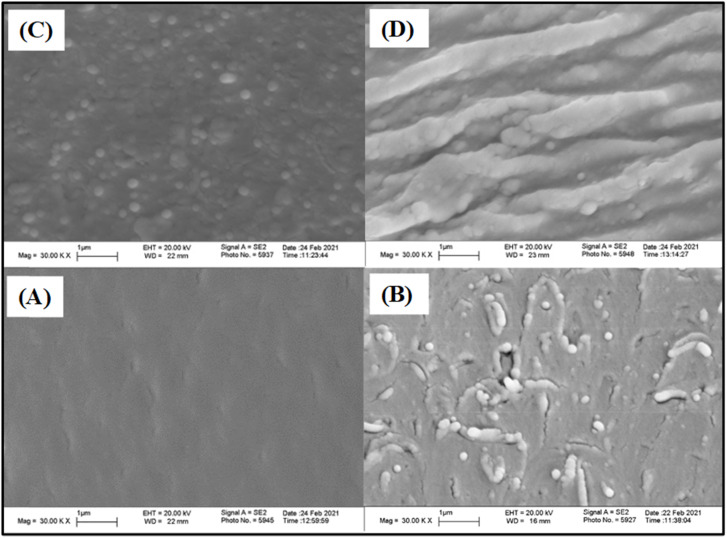
SEM images of the cured epoxy films with different CNT loadings; (A) EA-0, (B) EPBCNT-4%, (C) EAJ-0 and (D) ECB-CNT-4% composites.

The morphology of the epoxy composites was observed using SEM analysis. Fig. 7 shows the SEM images of the fractured surfaces of EA-0, EAJ-0, EPB-CNT-4% and ECB-CNT-4%. EA-0 exhibits a smooth surface due to the absence of filler, whereas discrete silica particles produced by the sol–gel reaction of APTS can be seen in EAJ-0.^33^ Compared to ECB-CNT-4%, the external diameter of MWCNT in EPB-CNT is lower due to physical bonding between the conjugated structure of CNTs and epoxy matrix.^34^ In ECB-CNT-4%, the CNT surface is diffused into the epoxy polymer chains. Therefore, the diameter of the tubes has increased due to the penetrated interface with the matrix and due to the hyperbranched siloxane-grafted MWCNT structure. The NH_2_/NH groups from GA-CNT have reacted through the epoxide ring of DEGEBA, and is well-bonded chemically to the polymer matrix. The CNTs are well-isolated and well-dispersed, and form a thicker network due to the hyperbranched (APTS-GPTS) grafting and polymer chains wrapping around their surface. The outer diameter of the original CNTs, which was around 10–20 nm as supplied, increased to 300–600 nm due to matrix imbibition and silane grafting^35^ Such a core–shell structure of epoxy polymer chain-bound CNT enhanced the transfer of stress from the matrix to the reinforcement, thus improving the mechanical properties of the composite material. The hydrolysed siloxane structure in GA-CNT became more condensed in the composite due to the increased pH caused by the addition of Jeffamine-D-400 to cure DEGEBA, which may also contribute to the composite's improved thermal stability.

### 3.5 Glass transition temperature (*T*_g_) and storage modulus (*E*′)

**Fig. 8:**
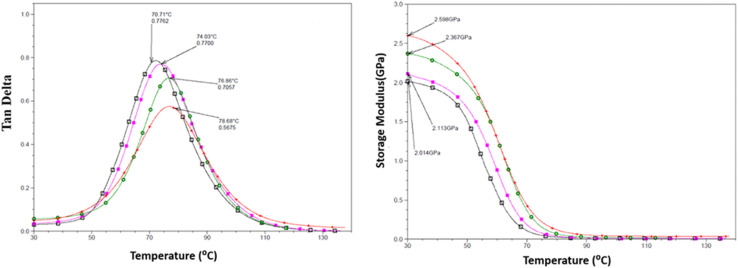
Temperature variation of tan *δ* (left) and the storage modulus (right) in the ECB-CNT Composites with wt%: 0 (□), 1(■), 2 (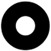) and 4 (●).

**Fig. 9:**
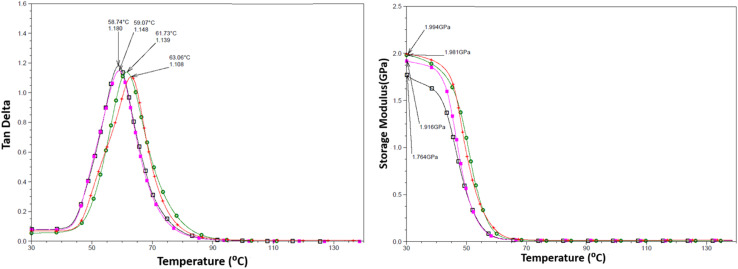
Temperature variation of tan *δ* (left) and the storage modulus (right) in the EPB-CNT Composites with wt%: 0 (□), 1(■), 2 (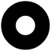) and 4 (●).

DMTA was performed to study the viscoelastic behaviour and to identify the glass transition temperature involving α-relaxation in the epoxy-CNT hybrid. The intensity of the tan *δ* curve indicates the viscous response of the material, measuring the energy dissipated as heat per cycle under deformation. This allows characterization of the reduction in the mobility of the epoxide network, as supported by relevant studies.^6,36,37^ The reinforcement effect of CNT on the temperature-dependent mechanical properties of both physically and chemically bonded nanocomposites (EPB-CNT and ECB-CNT) was investigated by comparing the storage modulus, glass transition temperature and tan *δ* values with those of neat epoxy in Fig. 8 and 9. This study shows that with the increase in temperature, the onset of segmental motion leads to a sharp increase at 58 °C in tan *δ* for pure epoxy (EJ-0). This peak corresponds to the α-relaxation temperature (*T*_g_). However, for composite films, the pristine CNT filler slightly reduces the segmental motion of epoxy chains in the EPB-CNT system due to the physical bonding of CNT to the epoxy matrix, whereas the hyperbranched siloxane-modified CNT in the ECB-CNT system massively reduces the segmental motion of the polymer chain due to chemical bonding. This results in stiffer films and a *T*_g_ shift toward higher temperatures. The considerable increment in *T*_g_ in the ECB-CNT composites is attributed to the increased interaction between the polymer matrix and the modified CNT filler at the interfacial zone due to increased surface area. The modified CNT structure provides greater interpenetration with the epoxy network and the mobility of the epoxy back bone was suppressed due to the increased interfacial interaction by the newly formed N–C bond between the NH_2_/NH groups of the GA-CNT and the epoxide group of DEGEBA, which contributes to the increased *T*_g_. Also, CNT modification could reduce both the agglomeration of CNT tubes due to inter-tube van der Waals interactions and the free volume between the tubes, which resulted in increased stiffness and therefore increased *T*_g_. The *T*_g_ for the EJ-0 is 58 °C, which increased to 71 °C in EAJ-0 due to silica inclusion from APTS. The increment with modified CNT reinforcement suggests that the mobility of the polymer chain is further restricted due to increased stiffness, which makes the polymer chains more rigid, increasing the *T*_g_ and damping the tan *δ*. The broader glass transition peak for higher CNT wt% composites and increased damping with increased CNT wt% can be explained by the energy dissipation from the matrix to the modified MWCNT reinforcement. In pristine composites, the pristine surface energy is much higher than that of the GA-CNT and the *T*_g_ shift is lower in the EPB-CNT composites (Table 1) compared to the ECB-CNT composites (Table 2). At 4 wt% CNT loading, the ECB-CNT composite *T*_g_ increased by 21 °C relative to the neat epoxy (EAJ-0) to 78.68 °C (Table 3), which is attributed to the interpenetrating effect of the chemically bonded GA-CNT structure in the epoxy matrix.

J. Macan *et al.*^6^ reported that the epoxy group of GPTS first reacts with primary amines, and there is less reaction with the secondary amine due to steric hindrances by the inorganic silica network, leading to a reduction in *T*_g_. However, Z. Ahmad *et al.* later proved the enhancement effect of the (GPTS-APTS)-modified silica on the glass transition temperature.^37^ In this study, we used a slight excess of APTS to ensure the availability of free secondary/primary amines after reacting with GPTS, allowing further reaction with the epoxide ring of DEGEBA. This provides the interpenetrated filler -matrix network and enhanced interfacial interaction, and thus considerable improvement in the mechanical properties of the hybrid material. The solvent DMAC significantly reduced the viscosity of the system during the sol–gel process, and thus increased the dispersion.

**Fig. 10:**
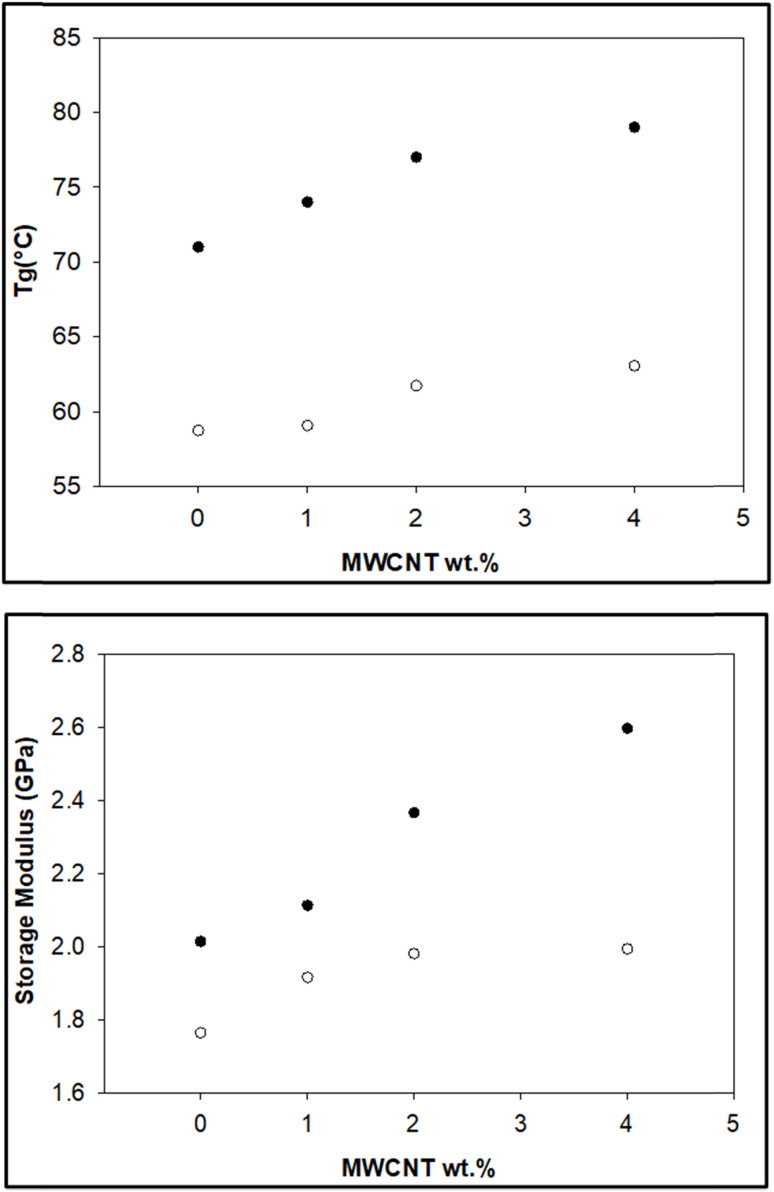
Variation of *T*_g_ (°C) (upper) and storage modulus (lower) in ECB-CNT (●) and EPB-CNT (○) composites with MWCNT wt%.

The storage modulus is a measure of a material's elasticity. The temperature variations of the storage moduli for the two composites are shown in Fig. 8 and 9. The storage modulus depends on the type of bonding, and gradually increases with increasing concentration of modified CNT. At 30 °C in the glassy region, the storage modulus (*E*′) of EAJ-0 is 1.76 GPa. This value increases slightly with the addition of CNT, which is in line with the increasing CNT wt% in the EPB-CNT system. In ECB-CNT hybrids, the modification of MWCNT with two coupling agents and the silica network formation did not affect the viscosity or the cross-link density of the hybrid system due to optimum usage of the solvent. However, it helped to attain the required stiffness, and thus the increment in the storage modulus with increased GA-CNT wt%. As temperature increases, the storage modulus drops by about an order of magnitude in the elastic region for all films. It approaches the *Y*-axis around 70 °C, and remains almost the same in the rubbery plateau region. DMTA analysis revealed that GA-CNT addition significantly reduces the mobility of the polymer–CNT networks, developing a more homogeneous cross-link topology, which is finally converted to a significant increment in *T*_g_ in the case of the ECB-CNT composites relative to EAJ-0 and EPB-CNT system. Fig. 10 gives a comparison of both *T*_g_ and storage modulus with MWCNT wt% for both EPB-CNT and ECB-CNT systems.

### 3.6 Thermal stability

**Fig. 11:**
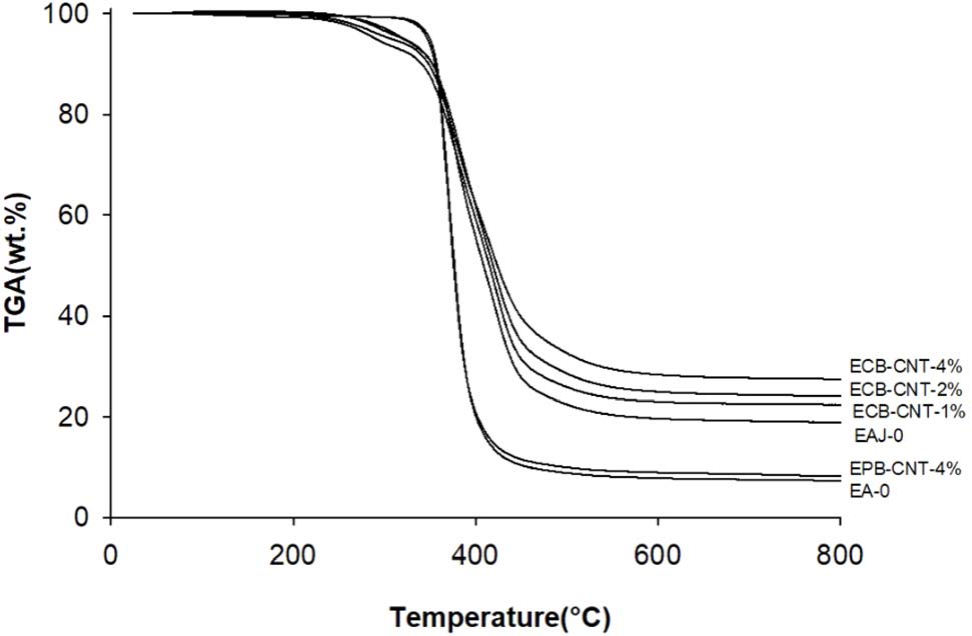
TGA thermograms of different epoxy-CNT composites.

Fig. 11 shows the thermal degradation behaviour of the neat epoxy film, EPB-CNT-4% and ECB-CNT hybrids with 1%, 2% and 4% GA-CNT. The primary decomposition of the prepared composites occurs in the temperature range of 369–385 °C, corresponding to the degradation of bisphenol-A groups. The thermal decomposition temperature (TDT) of EJ-0 is 369 °C, and it is slightly increased in ECB-CNT-4% due to the inclusion of pristine CNTs. In EAJ-0, TDT increases by 8 °C. Meanwhile, in the ECB-CNT composites, the decomposition temperature increases further due to the enhanced interfacial interaction between the modified CNT and the polymer matrix. The TDT and char yield of the chemically bonded composites increase significantly as a result of silanization and the resulting silica network.^21,36^ Minjae Kim *et al.*^38^ studied the effect of CNT-reinforced epoxy composites, and found that silanization of CNTs with GPTS is more effective in improving the thermal stability of the epoxy composites. Similarly, in our study, we achieved increased thermal stability using GPTS silanization, followed by APTS modification of –COOH MWCNTs.

In the ECG-CNT composites, the initial decomposition occurs in the temperature range of 200–400 °C, which is attributed to the decomposition of bonded silanes to MWCNTs. The TGA overlay in Fig. 11, showing different GA-CNT concentrations in the epoxy matrix, clearly illustrates the significant effect of the modified CNTs on the thermal stability as the CNT concentration increases.

### 3.7 Tensile analysis

**Fig. 12:**
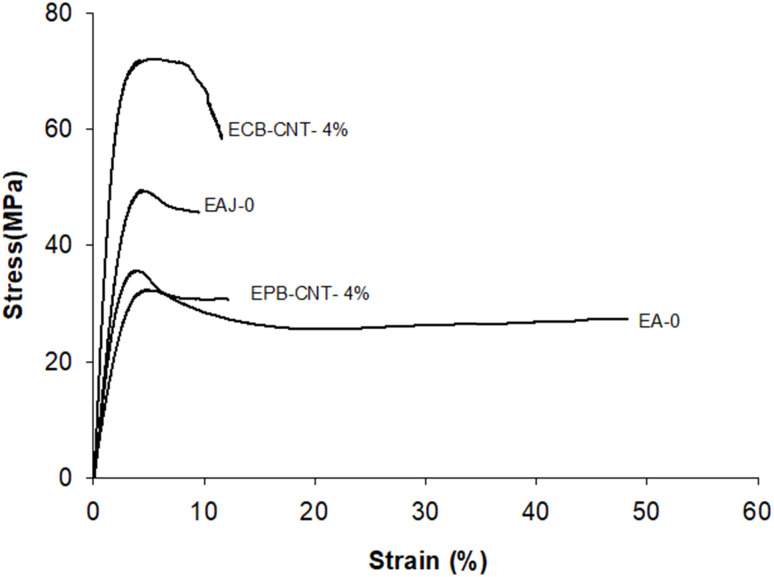
Tensile stress strain curves of different epoxy-CNT composites.

The mechanical properties (*e.g.*, tensile strength, tensile modulus and strain) for EJ-0, EAJ-0 and hybrid films with 4% CNTs from both physically bonded and chemically bonded systems were studied by tension testing at room temperature. All polymer-CNT systems have some requirements, like interfacial adhesion between the matrix and the fillers, aspect ratio, good dispersion, alignment, interfacial stress transfer and purity of the tubes. CNTs must be uniformly dispersed in the polymer matrix to achieve more uniform stress distribution, and minimize the presence of stress concentration centres due to aggregation. Alignment of the tubes is also necessary to maximize the strength and stiffness. Stiffness alone is not beneficial beyond the optimum point. In the EPB-CNT films, there was a greater chance of settling of the dispersed CNTs compared to that ECB-CNT systems. The viscosity of the polymer solution and the cross-linked network structure in the ECB-CNT system keep the tubes dispersed in the polymer matrix even in the drying step. Sonication of the CNT solution also must be controlled to avoid the breaking of ECB-CNT. This bonding between the GA-CNT and epoxy matrix will reduce the void formation due to CNT aggregation, and thus leads to successful interfacial stress transfer from the matrix to the filler in the case of the ECB-CNT film. The chemical bonding increases the stiffness, ensuring a higher tensile strength and modulus compared to the same weight percentage of pristine CNT systems. The stress–strain curves for EJ-0, EPB-CNT-4%, EAJ-0, and ECB-CNT-4% are given in Fig. 12 and the tensile properties are summarized in Table 4. Pristine CNT provides a small improvement in the tensile properties.^38^ In our study, the tensile modulus of ECB-CNT-4% increased to 1.71 GPa from 1.11 GPa, which is the tensile modulus of EJ-0. As can be seen in the table, ECB-CNT-4% exhibited a profound increment in the tensile properties compared with the film without any reinforcement. This enhancement of the mechanical properties is due to the improved load transfer efficiency from the epoxy matrix to the reinforcement and the reduced CNT aggregation. The sol–gel structure, along with the absence of aggregation and voids in the matrix, provides additional resistance to fracture under tensile load. Alireza Yaghoubi *et al.*^39^ tested the effect of silanization of MWCNT on the tensile properties in the polyurethane matrix, and explained that the increment in the cell density is due to silanized CNT inclusion in the matrix, as shown by SEM pictures. In Fig. 7, the SEM image of the composite with pristine CNT clearly reveals the void formation due to less interfacial interaction created from the physical bonding between the matrix and the CNTs. The obtained results also agree with previous studies,^40^ which show that the tensile properties of the polymer-CNT composites are highly sensitive to interfacial interaction and load transfer between the polymer matrix and the CNT reinforcement.

**Table 4:** Tensile properties of physically and chemically bonded epoxy-MWCNT composites with 0 and 4 wt% MWCNTs

CNT wt%	Tensile modulus (GPa)		Tensile strength (MPa)		Strain at break (%)	
	EPB-CNT	ECB-CNT	EPB-CNT	ECB-CNT	EPB-CNT	ECP-CNT
0	1.11	2.02	32.3	49.38	48	9.48
4	1.71	3.94	35.6	72.02	12.05	11.6

## 4. Conclusions

The epoxy composites have been prepared from diglycidyl ether of bisphenol-A (DGEBA) using Jeffamine-D-400 as a curing agent for films, where CNTs are physically bonded within the network of EPB-CNT composites. In ECB-CNT composites, 50% of the curing was done using APTS, followed by the remaining 50% with Jeffamine-D-400, resulting in covalent bonding of the CNTs with the polymer network. Silanization of CNTs was first performed with GPTS, and then the CNTs were reacted with APTS through sol–gel processing. The combination of GPTS and APTS, along with their mutual reactions during hydrolysis and condensation in the sol–gel process, resulted in a modified CNT network within the epoxy matrix of the chemically bonded system. The structure and morphology of the hybrid films with different %s of modified CNT ranging from 0 to 4 wt% CNT have been analysed. The successful modification of CNTs and effective curing of the epoxies were studied using FTIR. Thermal stability and the mechanical properties of the composites were fortified with the inclusion of modified CNT. The TGA, DMTA, and tensile properties support the increased interfacial interaction due to the silanization of CNTs and the resulting cross-linked network structure in the ECB-CNT composite. SEM and AFM results also indicated the formation of an interface, and increased interactions between the polymer matrix and the filler in the ECB-CNT composite films due to the merging of surfaces. Thus, the viscoelastic properties of the compatibilized composites increased with the amount of GA-CNT. The present study attributes the significant dampening in the tan *δ* curves and rise in *T*_g_ to enhanced adhesion caused by a stiffer interphase between the matrix and the silica network. This enhancement not only improves the thermomechanical characteristics, but also protects the organic matrix against thermo-oxidative damage at higher temperatures. Thermoset composites with enhanced mechanical strength, adhesion, and heat resistance are important for use as high-performance coatings on metal surfaces. Additionally, this technique could offer insights into the widespread production of high-performance CNT composite materials, while ensuring safe procedures.

## Conflicts of interest

There are no conflicts to declare.

## Supplementary Material

RA-014-D4RA06831A-s001
