# Supplementary material for: Enhanced thermomechanical properties of epoxy-multiwalled CNT nano-composites
Source: RSC Adv. 2024 Nov 5;14(48):35360–72. doi: 10.1039/d4ra06831a (PMC11536937; doi:10.1039/d4ra06831a)
Supplement: RA-014-D4RA06831A-s001 [file RA-014-D4RA06831A-s001.pdf]

Supplementary Information (SI) for RSC Advances.
This journal is © The Royal Society of Chemistry 2024

Preparation of Epoxy-Multiwalled CNT Nano-Composites and the enhancement of thermomechanical properties by incorporating modified MWCNT as reinforcing material.

Abdullah Alhendal[1]*

[1]Department of Chemistry, Kuwait University, P. O. Box 5969, Safat, 13060, Kuwait.
**Corresponding author Email: Abdullah.alhendal@ku.edu.kw.*

Table 1S. FT-IR frequency of epoxy vs cured epoxy.

Table 2. FTIR frequency of epoxy vs cured epoxy

Wavenumber cm−1	Assignments
3437	N–H stretching
3057	C–H stretching
3037	C–H stretching
2966	Asymmetrical C–H stretching CH3
2928	Asymmetrical C–H stretching CH2
2882	Aldehyde C–H stretching
2872	CH2 sym. & asym stretching
1740	C=O stretching
1610	N–H bending
1582	N–H bending
1513	Deformation vibration mode of CH3
1460	C–H deformation, CH2
1385	Symmetrical C–H deformation, CH3
1362	C–O stretching
1298	C–H in plane bending
1246	C–H in plane bending
1084	C–H in plane bending
1035	C–H in plane bending
975	C–H out of plane deformation
914	Epoxide ring
832	C–H out of plane deformation
560	O–H out of plane vibration
